# Local Delivery of Growth Factors Using Coated Suture Material

**DOI:** 10.1100/2012/109216

**Published:** 2012-05-15

**Authors:** T. F. Fuchs, C. Surke, R. Stange, S. Quandte, B. Wildemann, M. J. Raschke, G. Schmidmaier

**Affiliations:** ^1^Department of Trauma, Hand and Reconstructive Surgery, University Hospital Muenster, Waldeyerstrasse, 48149 Muenster, Germany; ^2^Julius Wolff Institut for Biomechanics and Musculoskeletal Regeneration, Charité, Berlin University Hospital, Augustenburger Platz, 13353 Berlin, Germany; ^3^Department of Orthopedics and Traumatology, Heidelberg University Hospital, Im Neuenheimer Feld, 69120 Heidelberg, Germany

## Abstract

The optimization of healing processes in a wide range of tissues represents a central point for surgical research. One approach is to stimulate healing processes with growth factors. These substances have a short half-life and therefore it seems useful to administer these substances locally rather than systemically. One possible method of local delivery is to incorporate growth factors into a bioabsorbable poly (D, L-lactide) suspension (PDLLA) and coat suture material. The aim of the present study was to establish a procedure for the local delivery of growth factors using coated suture material. Sutures coated with growth factors were tested in an animal model. Anastomoses of the colon were created in a rat model using monofilament sutures. These were either untreated or coated with PDLLA coating alone or coated with PDLLA incorporating insulin—like growth factor-I (IGF-I). The anastomoses were subjected to biomechanical, histological, and immunohistochemical examination. After 3 days the treated groups showed a significantly greater capacity to withstand biomechanical stress than the control groups. This finding was supported by the results of the histomorphometric. The results of the study indicate that it is possible to deliver bioactive growth factors locally using PDLLA coated suture material. Healing processes can thus be stimulated locally without subjecting the whole organism to potentially damaging high systemic doses.

## 1. Introduction

In spite of considerable progress in operating techniques, the use of histocompatible materials, and minimally invasive operating procedures, healing processes following surgery are frequently disturbed. This means that the optimisation of healing processes in a wide variety of tissues is a central topic for surgical research. Healing processes can be upset by many different factors such as prior illness, old age, inadequate nutrition, immune suppression, or sepsis. Current initiatives in surgical research are therefore seeking methods by which healing processes can be stimulated.

The use of growth factors opens up an entirely new range of possibilities for stimulating healing processes and has demonstrated to stimulate cell growth in a variety of tissues [1–5] that could lead to further improvement of healing processes and reduction of complication rates. Shorter hospital stays and financial savings would be another aspect. Further positive side effects would be the reduction of (a) nosocomial infections, (b) pneumonia resulting from immobilisation, and (c) deep leg vein thrombosis with the associated risk of lung embolisms (lethality 5%).

Growth factors are small proteins which act as mediators within cells and thus influence cell division, growth, differentiation, and protein synthesis. They are present in plasma at extremely low concentrations and probably regulate all organ systems.

So far growth factors have been used systemically [6], either by the additional implantation of carriers in the form of collagen sponges [7], with catheters, by injection, or in nutritional supplements [8, 9]. These techniques yielded demonstrable effects on the healing of bone, cartilage, and tissues in the gastrointestinal tract, but they did not appear practicable for clinical use. It seems useful to concentrate efforts on developing suitable systems for delivering them locally without subjecting the whole organism to high systemic doses.

Such local delivery systems need to enable the substance to act and to ensure a sufficient level of biological activity for optimal effects. The carrier materials must be bioabsorbable [10] and biocompatible to minimize immune reactions, toxicity, and side effects. Neither locally induced inflammatory reactions nor physical blocking as a result of incomplete metabolism can be allowed to inhibit the healing process. The carrier materials must be sterilizable and permit flexibility in the amounts of the substances being delivered. The incorporated substances need to be released in a continuous and controlled manner so that they are not absorbed before exerting their effects. A high level of user friendliness and easy handling are also desirable for the operating surgeons [10].

A procedure has therefore been developed and brought into use that turns implants into drug carriers. Using these implants, substances can be placed where required and released in a controlled manner without causing systemic stress to the organism. A biodegradable surface coating based on poly (D, L-lactide), PDLLA, was developed for this purpose. Bioactive substances such as growth factors can be incorporated into this coating [11, 12].

Poly  (lactides) and poly(glycolic acids) and their co-polymers are widely available biomaterials and can be used as drug carriers [10, 11, 13]. The properties of the PDLLA coating developed were investigated in *in vitro* studies. These included the thickness of the layer (about 10 *μ*m), its firmness, its decomposition dynamics *in vivo *and *in vitro*, the release kinetics of the incorporated growth factors, and their stability in the coating.

One of the most frequently used procedures for the reconstruction of damaged tissue is suturing. We set out to develop a procedure for coating suture material with growth factors without altering properties such as absorbability, tissue tolerability, and handling.

Suture insufficiency following intestinal resection is one of the most severe complications in gastrointestinal surgery. The rate of clinically significant anastomotic insufficiency following colon resection is still between 3.4 and 8% [14, 15] in spite of progress in anastomosis techniques. In the clinical setting suture insufficiency with development of peritonitis is diagnosed primarily in the early postoperative phase (day 1–day 10) and leads to abscesses and fistulae. In various experimental models, the positive influence of endogenous growth factors on wound healing has been shown [6, 14–18]. The biological actions of these growth hormones include mitogenic [4, 18, 19] or chemotactic effects [20], release of cytokines [4], stimulation of angiogenesis [4] and improvement of extracellular matrix production [21]. Systemic keratinocyte growth factor (KGF) or insulin-like growth factor 1 (IGF-1)—shown experimentally—leads to improve anastomosis healing in rat colon [6, 18]. However, as giving an excess of growth factors can have potentially harmful effects on the whole organism, methods should be investigated by which growth factors can be applied at defined target areas in defined quantities. A newly developed technology makes this possible. The carrier material used is a biodegradable PDLLA coating [11, 12]. The aim is to improve the healing of anastomoses following colorectal resection in an animal model in order to reduce the rate of occurrence of anastomotic insufficiency and its sometimes catastrophic consequences.

## 2. Material and Methods

### 2.1. Coating

In the process described here a bioabsorbable polylactide coating PDLLA is applied to suture material in a “cold" coating process to render incorporated substances “biological active.” This process turns the implant itself into a drug carrier.

The coating process takes place under clean room conditions at room temperature and therefore also allows thermolabile substances to be incorporated without affecting their biological activity [22]. The modifiable coating thickness was investigated using scanning electron microscopy. The biomechanical strength of coated and uncoated suture material was tested by a load to failure test using a material testing machine (Zwick).

For the rat anastomosis model, Ethicon 6/0 PDS sutures (45 cm long) were coated with 30 *μ*g rh-IGF-I per suture.

An *in vitro *elution test was carried out on suture materials over a period of 10 days. Aprotinin was used in this elution test. Aprotinin is a protein with a similar molecular weight (approximately same size) as IGF-I.

### 2.2. Experimental Animals

Anastomoses were performed in 5-month-old female Sprague Dawley rats. Up to 24 h before and immediately after the operation, they had free access to water and dry feed. The animal experiment was carried out with the permission of the responsible authority (*Landesamt fur Arbeitsschutz, Gesundheitsschutz und technische Sicherheit Berlin*—Berlin state office for technical safety and the protection of work and health), permit number G0045/01.

### 2.3. Operation

Dry feed was replaced with a special fibre-free feed 24 h prior to the operation. The operation was performed under general anaesthesia. Initial inhalation narcosis used a mixture of oxygen, nitrous oxide, and isoflurane. A mixture of ketamine hydrochloride (80 mg/kg body weight) and 2% xylazine (12 mg/kg body weight) was then given by intraperitoneal injection.

This anaesthetic mixture narcotised and anaesthetised the experimental animals for about 90 minutes.

Blood samples were taken from the retrobulbar venous plexus of the right eye. About 1.5 mL of whole blood was taken for the determination of serum and blood parameters. Prior to the operation, the anaesthetised animals were weighed and their rectal temperature was determined.

Each rat's abdomen was shaved and then depilated. After skin disinfection, a longitudinal laparotomy was performed. The intestine was exposed under an operating microscope up to the transverse colon, which was cut 1 cm distal to the hepatic flexure. An end-to-end anastomosis was then created using a continuous seromuscular suture with randomised use of either coated or uncoated sutures. The anastomosis was tested for leaks and luminal continuity prior to layered closure of the abdominal wall (see Table 2).

Of the 16 animals in each group, 8 were subjected to biomechanical evaluation and 8 to histological/immunohistochemical evaluation.

Follow-up observation was carried out for 24 h. Blood samples were taken, and body core temperature was measured at day 3 and again at day 7 after surgery. The anastomosis of the colon was evaluated biomechanically, histomorphometrically, and immunohistochemically.

### 2.4. Biomechanical Evaluation

Biomechanical evaluation took the form of an intraluminal pressure stress test. For each time point, *n* = 8 animals were investigated. The anastomosis was subjected to stress using isotonic saline solution delivered by a 4-roller pump. At the same time, the intraluminal pressure at the level of the anastomosis was derived using a force transducer (Mammendorfer Institut). The data were amplified and transferred to a computer with the aid of the measurement data analysis programme Catman. The maximum bursting pressure was evaluated in each case (Figure 3). Eight animals per group were evaluated for each time point. Statistical comparisons were made using the nonparametric Wilcoxon-Mann-Whitney test as the data for the individual groups were independent and not normally distributed. The statistical software SPSS was used. 

### 2.5. Histomorphometric and Immunohistochemical Evaluation

After the animals had been killed (*n* = 8 per group for each time point), the anastomosis was dissected out. It was separated 1 cm proximally and distally, and the tissue was fixed. HE and Van Gieson stains were used for the histological evaluation. To analyse the inflammatory reaction and angiogenesis, immunohistochemical investigations were performed using CD68 antibodies, alpha smooth muscle actin, and factor VIII stain smooth muscle cells and endothelial cells. 

Evaluations were randomised and carried out blindly. In the area of the anastomosis, the connective tissue bridging and reepithelisation were evaluated. Vascularisation was also assessed and allocated a score. Inflammatory cells marked with CD68 antibodies were also counted in the area of the anastomosis. Reepithelisation was taken to be an indication of functioning replacement tissue in the area of the anastomosis.

Granulation, reepithelisation, connective tissue bridging, and revascularisation, as well as numbers of fibroblasts, necroses, and macrophages, were evaluated by a modified score published by several authors [18, 23–25].

Tissue reaction (score):

1. no or minor reaction
2. poor reaction
3. mean reaction
4. strong reaction

Statistical evaluation was carried out using the Kruskal Wallis test and the Mann-Whitney *U* test and later balanced using a Bonferroni-Holmes adjustment. The significance level was taken to be 0.05.

## 3. Results

### 3.1. Coating

**Figure 1:**
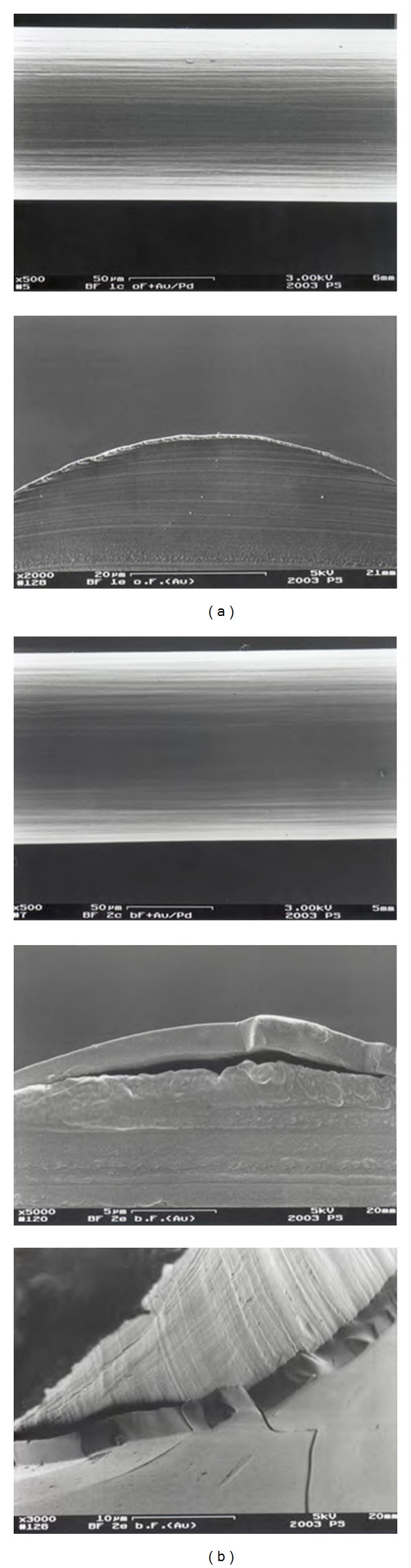
SEM images of uncoated (a) and coated (b) monofilament sutures. The coating forms an smooth even layer of 10 *μ*m thickness in which growth factors can be incorporated*. In vivo *the PDLLA coating is hydrolyzed and the growth factors are released in their biological active form.

**Figure 2:**
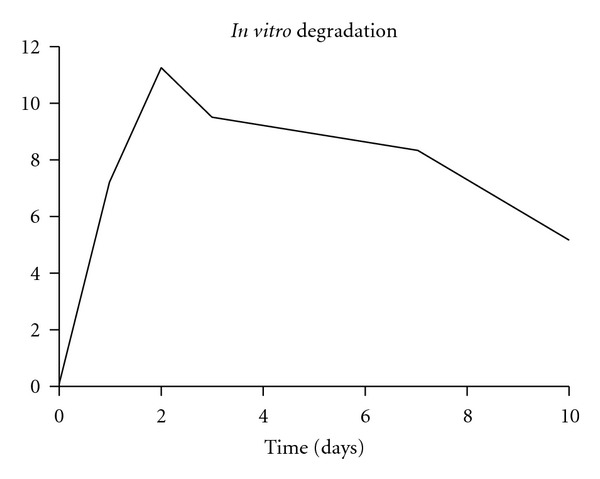
For *in vitro *release tests aprotinin was used. aprotinin is a protein with approximately same size as IGF-I. The aprotinin concentration was measured over the time. After an initial peak, a constant release over at least 10 day took place.

**Figure 3:**
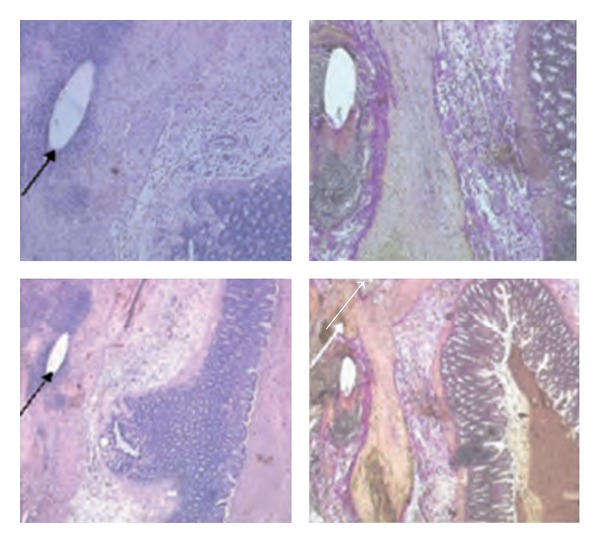
For histologic examinations, the region of the anastomosis was dissected 2 cm proximal and distal of the anastomosis. HE stains and von Gieson stains were done to evaluate the anastomotic healing. The black arrow marks the suture material, and the white arrow marks the anasotomosis.

Examination using a scanning electron microscope showed the PDLLA layer on the sutures to be smooth and even and about 10 *μ*m thick (Figure 1). In the elution test, the proteins incorporated into the coating were released at a constant rate over 10 days after an initial peak (Figure 2) The biomechanical stress test showed no significant difference between the coated and uncoated sutures (*n* = 5 per group).

### 3.2. Clinical Evaluation

No differences in body temperature or weight were found at any time in any of the groups. Inflammatory cell counts were normal throughout.

### 3.3. Biomechanical Evaluation

**Figure 4:**
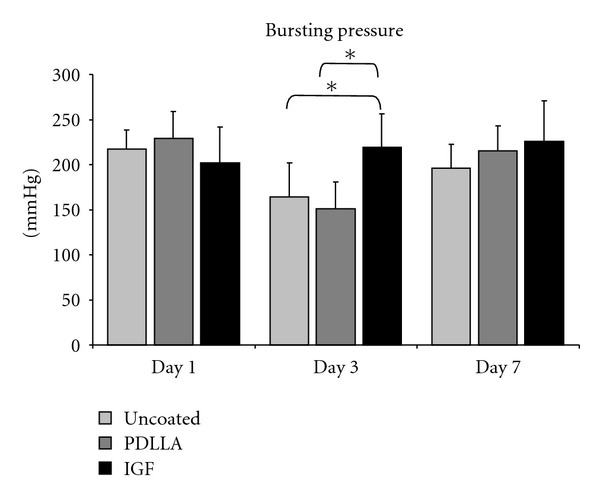
Statistical analyses of the bursting pressure tests showed a decrease of stability three days after surgery in the control groups (uncoated and PDLLA coated). This decrease was not seen in the IGF-I-treated specimens. At day three after surgery, a significant higher bursting pressure was measured in this group compared to the controls. 7 days after surgery no significant difference was seen in between the groups. Statistical analyses were performed using ANOVA analyses and were adjusted with the Bonferroni test (*P ≤ 0.05).

No significant differences in bursting pressure were found between the groups 24 hours after the operation (data not shown). However, three days after the operation, the stability of the anastomosis in group III (PDLLA + IGF-I) was significantly higher than in the control groups I (uncoated sutures) and II (PDLLA coated sutures). After seven days the animals treated with coated sutures impregnated with growth factors showed greater biomechanical stability in the area of the anastomosis than the control groups, but this difference did not achieve significance. The loss of stability in the control groups 3 days after the operation did not occur in the animals treated with IGF-I. The bursting pressures reached after 3 days in the growth-factor-treated animals were reached by the PDLLA group only after 7 days. The anastomoses treated with uncoated sutures did not reach this level of stability at all during the experiment (Figure 4).

### 3.4. Histomorphometric and Immunohistochemical Evaluation

**Figure 5:**
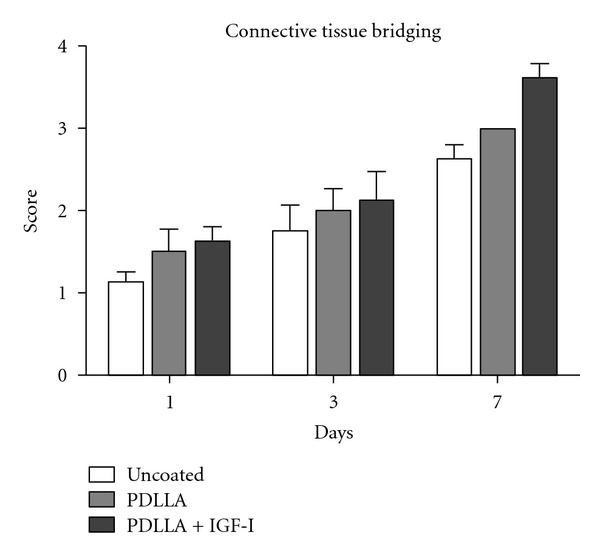
Collagen synthesis increased over the period of time and therefore the amount of cellular connective tissue. Looking at the connective tissue bridging of the anastomosed region over the time, an increased level of connective tissue in the IGF-I-treated group became obvious, even though there was no significant difference measurable at day one and three but at day 7. This effect due to locally applied IGF-I might explain the increased mechanical stability at day three after surgery, since the increase of connective tissue was significantly higher in the growth factor treated group in between day one and three, but was not significant in the control groups.

Collagen synthesis increased over the period of time. Looking at the connective tissue bridging of the anastomosed region over the time, an increased level of connective tissue in the IGF-I treated group became obvious, even though there was no significant difference measurable at day one and three but at day 7. This effect due to locally applied IGF-I might explain the increased mechanical stability at day three after surgery, since the increase of connective tissue was significantly higher in the growth-factor-treated group in between day one and three, but was not significant in the control groups (Figure 5).

**Figure 6:**
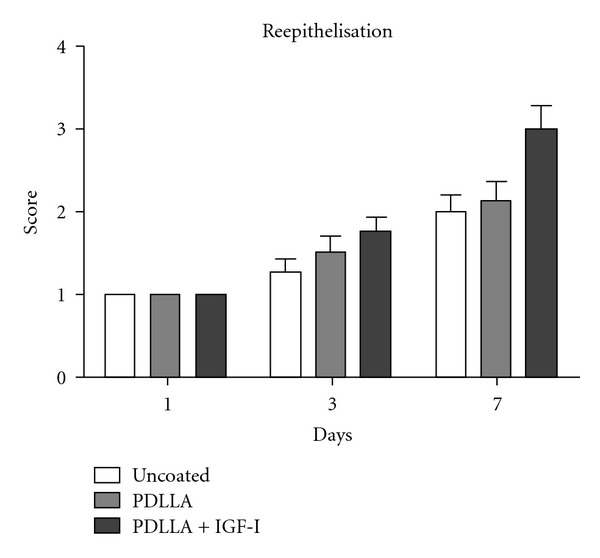
Reepithelisation of the mucosa became obvious especially at day seven after surgery in all groups, and a tendency of advanced reepithelisation was seen at this time point in the IGF-I-treated group. In this group the increase of reepithelisation became significant already in between days one and three and three and seven, whereas a significant difference in the control groups not until day seven after surgery occurred.

Reepithelisation of the mucosa became obvious especially at day seven after surgery in all groups, and a tendency of advanced reepithelisation was seen at this time point in the IGF-I treated-group. In this group the increase of reepithelisation became significant already between day one and three and three and seven, whereas significant differences of reepithelisation in the control groups did not become obvious until day seven after surgery (Figure 6).

**Figure 7:**
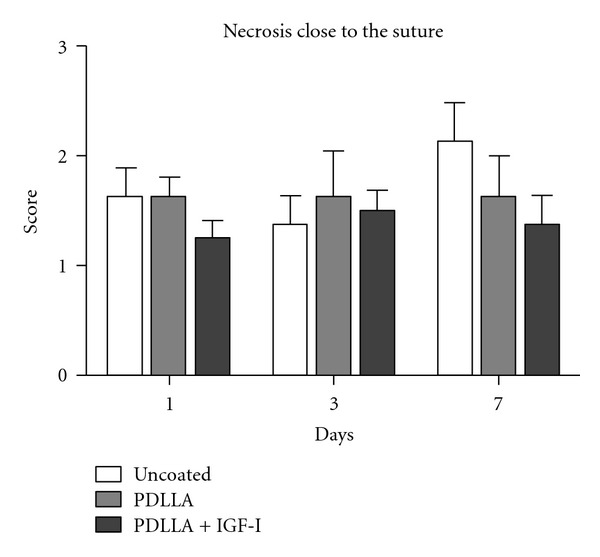
Except in the uncoated group in the region of the anastomosis at day three and day seven, necroses were seen rarely. Especially the coating and the applied IGF-I did not cause any side effects which lead to disturbance of the surrounding tissue at any time point.

Except in the uncoated group in the region of the anastomosis at day three and day seven, necroses rarely were seen. (See Table 1) Especially the coating and the applied IGF-I did not cause any side effects, which lead to disturbance of the surrounding tissue at any time point (Figure 7).

## 4. Discussion

Coating of medical devices such as coronary stents [26, 27] and catheters [28] or orthopaedic devices such as prostheses [29, 30] and intramedullary nails [31] becomes of more and more interest in modern medicine. Several studies describe coating of suture material [32, 33] either to prevent infection or to stimulate healing processes. Korenkov et al. applied TGF-*β*1 into the abdominal wall to augment their strength in rat hernia model, but there was no proof in increasing strength in the TGF-*β*1-treated groups [34].

This study was able to show that it is possible to coat suture material with growth factors and keep them biologically active after they are released out of the coating. It could be shown that local application of IGF-I can improve significantly the healing of intestinal anastomoses in the rat model. As well as ensuring the mechanical stability of the anastomosis, coated suture material can thus function as a drug carrier at the same time.

In our study we could show that coating suture material with a bioabsorbable polylactide provides an opportunity of delivering drugs locally in effective concentrations. This method is appropriate for a variety of indications and does not alter the proven properties of the surgical suture material.

At the same time, no side effects in means of disturbing healing processes caused by the polylactide-coating were seen. It even seems that using PDLLA coated sutures avoids the occurrence of necrosis.

The rate of anastomotic insufficiency following colon resection is between 3.4 and 8% [14, 15]. This rate of occurrence could be reduced when using coated suture material. Catastrophic consequences such as peritonitis and the resulting of additional operations and long stays in hospital could be reduced. Side effects of locally applied IGF-I were not seen in this study.

It has been shown that intraperitoneal or intracolic delivery of IGF-I or KGF can lead to improved healing of colonic anastomoses in an animal model [6, 18]. However, it has not yet been possible to achieve limited local delivery of growth factors. It has been found that expression of transforming growth factor (TGF)-*α* and vascular endothelial growth factor (VEGF) is increased in carcinoma of the colon [35]. In the light of this finding, it is particularly important to limit release in terms of both time and space. The use of coated suture material could offer an innovative means to achieve this. It opens up a new range of prophylactic options for complications associated with anastomoses in patients with reduced healing capacity, such as those with CED, or in patients receiving immune suppression following transplants or had radiation before.

In summary, the coating process described here should be seen as a key technology. The coating can be applied to a wide variety of implants made from different materials. The appropriate drug can be delivered locally in a targeted and individually tailored dose to treat the particular indication or pathogenic spectrum. The method can be used either to avoid giving high systemic doses, which may have negative side effects, or to provide local support for the systemic action of substances such as antibiotics.

The use of coated implants is still some way from being a standard procedure in surgery. However, the studies that have so far been performed and initial clinical experience are promising. Together with progress in the development of implants and operating techniques they could help to further reduce the occurrence of complications in surgery.

## Figures and Tables

**Table 1:** Analyses of the necrotic tissue reaction at the anastomoses in between the groups at day 1, 3, and 7 after surgery. Significant less necroses were seen at day 3 and 7 in the IGH-I-treated group.

Necroses	Day 1	Day 3	Day 7
Control group	1,0 ± 0,0^#^	3,4 ± 0,7	3,0 ± 0,9^#^
PDLLA group	1,4 ± 0,7	1,8 ± 0,7*	2,0 ± 1,3
IGF-I group	1,3 ± 0,7	2,0 ± 1,1*	2,0 ± 1,1

*P < 0.05 in between the control and PDLLA group; control and IGF-I group at day 3;

^#^ *P* < 0.05 within the control group in between days 1 and 3; days 1 and 7.

**Table 2:** Experimental  groups.

Group I	uncoated sutures	*n* = 16
Group II	PDLLA-coated sutures	*n* = 16
Group III	PDLLA + IGF-I-coated sutures	*n* = 16
